# A multi-class framework for fish species classification using deep learning technique

**DOI:** 10.1371/journal.pone.0342901

**Published:** 2026-02-12

**Authors:** Zain Farooq, Muhammad Ramzan, Muhammad Bilal, Muhammad Attique, Tae-Sun Chung, Anam Naz

**Affiliations:** 1 Department of Computer Science, Faculty of Computing and Information Technology, University of Sargodha, Sargodha, Pakistan; 2 Department of Software Engineering, Faculty of Computing and Information Technology, University of Sargodha, Sargodha, Pakistan; 3 Department of Software Engineering, National University of Computer and Emerging Sciences, Islamabad, Pakistan; 4 Department of Artificial Intelligence, Ajou University, Suwon-Si, South Korea; CCET: Chandigarh College of Engineering and Technology, INDIA

## Abstract

Fish species recognition is essential for ecological studies, fishery management, and marine biology. Accurate detection and categorization are critical for preserving biodiversity, allowing scientists to track species distribution, identify invasive species, and analyze the effects of environmental changes. The fish sector is essential to any country's food and agriculture. Identification of species by the morphology process is both inaccurate and costly. However, the manual process of measuring important details like species identification, length, and quantity is difficult to capture, which shows the need for automation. The merging of automated systems and artificial intelligence has revolutionized this industry. Recent advancements in image detection systems based on machine learning and deep learning have been explored across various domains. Yet, applying state-of-the-art deep model Convolutional Neural Networks (CNNs) to identify the fish species’ complexity of season and location, and limited public datasets pose a challenge for the detection. Machine learning and deep learning use artificial neural networks to simulate how humans think and learn, efficiently automating similar monitoring applications such as species identification on land and in water. You Only Look Once (YOLO) is a state-of-the-art method for object detection based on deep learning. The goal of this study is to develop a deep learning system for recognizing fish species using the YOLO paradigm. The Fish-Pak dataset, which includes information on tropical fish farming in Pakistan, consists of 915 images against 6 targeted classes, freely available at the Mendeley data source. To ensure the suggested YOLO architecture's improved performance on the Fish-Pak data collection, we will conduct an experimental comparison with other versions of YOLO v3 and V4. The total accuracy of fish species identification using the proposed methods is 99%, with an mAP of 99.65%, top performance results as compared to existing literature.

## Introduction

Fish are among the most diverse vertebrates on Earth and play a pivotal role in maintaining ecological balance and sustaining healthy aquatic systems. They provide essential protein and livelihood to millions, impacting nutrition, the economy, and employment globally through the activities of fisheries and aquaculture. Fish are key environmental indicators, and they must be studied and correctly identified to enable the conservation of biodiversity and the sustainable use of resources. Due to the rising population and climate change, the accurate observation and identification of fish are of growing importance for biological studies and fisheries management [1].

In recent years, the aquaculture sector has been characterized by rapid growth, diversification, and technological development, with a particular focus on the improvement of production efficiency and environmental sustainability [2]. Automation technologies, including those based on deep learning, computer vision, and machine learning [3,4], and transfer learning for fish identification [5], have enabled significant increases in productivity and operational accuracy [6,7]. In addition, the application of automated sorting and monitoring systems in fisheries could contribute to mitigating critical issues such as rising species demand and resource availability, which are at risk of being compromised by factors including population growth, climate change, and global warming [8,9].

The role of the fisheries sector in Pakistan is crucial in contributing to the food security and livelihood of the masses. It has accounted for 0.39% of the GDP in 2024–25. Although the sector is vital, it is plagued by major problems, including contamination, climate change, and the long-lasting impact of overfishing in the marine sector. Pakistan’s seafood sector is not living up to its potential with a 1,050 km coastline and miles of inland water, but the industry has been devastated by years of overexploitation, poor governance, and lack of investment in infrastructure such as cold chain and processing facilities [10], a recent policy brief underlines. Other stresses, such as increasing water temperature, salinity intrusion, and changing flow patterns, also further deteriorate aquatic habitats and decrease fish productivity [11]. However, with technological advances, institutional reforms, and a sustainable aquaculture strategy, Pakistan’s fisheries sector has tremendous potential for contributing towards national nutrition, earning foreign exchange, and increasing the income of the rural population.

Conventional methods for fish species identification are based on manual inspection and expert knowledge in taxonomy. But these methods are usually time-consuming, subjective, and susceptible to human errors, especially when processing large amount of data or underwater images that are incidentally influenced by illumination, occlusion and fish posture [12] These constraints indicate that traditional classification methods are insufficient in terms of scalability and speed and also point out the potential importance of developing automated, high-precision and intelligent fish classification algorithms with high robustness under various environments [13].

The contribution of this article is important since the automation of fish classification enables marine biologists, ecologists, and fisheries managers to better track aquatic biodiversity and to monitor marine resources in a more efficient way. Deep learning is a promising approach that can help to reduce the dependency on human experts and also enable the identification of species at a large scale, consistently and in real-time, supporting the sustainable fisheries and environmental protection [14]. The Pakistan fisheries area plays a vital role in food security and supports the livelihoods of many people. In the year 2024–2025, this sector contributed 0.39% to Pakistan’s GDP. This area also faces a few types of challenges, like pollution and climate change, but holds major potential [15]. A recent policy brief states that the seafood sector in Pakistan is underperforming despite a 1050 km long coastline and vast inland water systems because of decades of unregulated marine fisheries, overexploitation of stocks, ineffective governance, and insufficient investment in infrastructure (i.e., cold-chain, processing) [10]. Other acute challenges in the sector include pollution, an increase in sea temperature due to climate change, salinity intrusion, and variability in water flows, which worsen water habitat and consequently fish productivity [11]. Nevertheless, as technology is modernized, there is more regulation and sustainable expansion of aquaculture; this sector has significant potential to contribute to national nutrition, generate export income, and increase rural incomes.

In this work, we employ the Fish-Pak dataset, which consists of tropical fish in the following species: Mori, Silver carp, Rohu, Catla, Grass carp, and Cyprinus Carpio. The main objective is to construct a fish species classification system based on visual features, which can recognize more than one species per image. To tackle this, a YOLO-based architecture is introduced for fish detection and multi-class fish classification. This paper presents a study to investigate the state-of-the-art deep models, YOLO-V3 and YOLO-V4,  for training powerful fish recognition models. Overall, by studying accuracy, training speed, and detection efficiency, this work advances the automated underwater species recognition technology. The main contribution of the paper has been given as:

- Provides a detailed overview of existing state-of-the-art techniques for the detection of fish species.
- Exploration of diverse features for fish species detection using the proposed model.
- Investigation of state-of-the-art DL algorithms, such as CNN with YOLO architecture.
- Conducting comprehensive empirical analysis and performance evaluation based on various metrics.

The rest of the paper is structured as follows: “Related work” gives a comprehensive overview of related work concerning fish species classification with conventional and deep learning methods. The proposed method, the creation of the dataset, the architecture of the model, and the experimental setup are explained in “proposed research methodology”. In “results and discussion”, the results are discussed, and the model performance is evaluated, and in “conclusion and future Work”, we conclude the paper with suggestions for future work.

## Related work

Researchers have long been pledged to the challenging task of classifying fish species, facing difficulties due to the density of backgrounds and noise in images. Advanced methodologies have been employed over the years to accurately classify fish species in their underwater habitats [16]. This attempt increases the challenges, such as background noises, image distortion, and other complexities. Various studies have investigated innovative techniques like CNN, DL, ML, and Image Processing to enhance accuracy in fish species classification. The goal is to automate the process of recognizing and categorizing fish species, providing valuable insights into ecosystems and aiding conservation efforts [17,18]. Machine learning has been increasingly used in fish species classification from environmental data [19,20]. Several recent studies have explored different machine learning algorithms and techniques for detection. A novel approach [21] was introduced in hybrid models that combine supervised machine learning algorithms with feature extraction techniques to enhance the classification accuracy of submerged images of 9 classes of sea fish species, utilizing dimensionality reduction techniques using a random forest model, achieving results of 99.2% with the feature extraction method. Another framework was developed [22] for ML-based image collection, labeling, and classification for species detection, targeting to facilitate AI applications. The framework demonstrates its potential for automating fish class detection from images uploaded to the application. The detection tool [23] used in the research consisted of multispectral data captured, focusing on three fish species: horse mackerel, Atlantic mackerel, and sardines. ML algorithms are applied to automatically discriminate between species, capturing small 5x5 pixel regions of the fish to generate spectra for classification. Three algorithms: KNN, Multilayer Perceptron [MP], and SVM, are compared for their classification accuracy. The best classification accuracy achieved was 63.8% using the SVM model. Furthermore, another study investigated that the Fish-Pak dataset is used to build a deep learning-based fish species classification framework which consists of a 32-layer CNN based on modified VGGNet model inclusion of pre-trained model of VGG-16, AlexNet, GoogLeNet and ResNet-50 in terms of their classification performance and found superior. The study attained high accuracy, though it has inherent limitations of preprocessing techniques variations in lighting, occlusions, and background noise may make the model not work well in real underwater conditions. Moreover, the ability to make an extensive comparative analysis of the classification efficiency in real-time is absent in the study. However, this study shows that the model proposed is good at extracting visual features for classification. However, enhancing the applicability of the model includes transfer learning with larger datasets, robustness testing in different environments, and model optimization for real-time usage [24].

The small pelagic fish classification for the detection of fish species was employed and consists of a classifier using morphological and positional features as input to extract features from data to combine with other features. The methodology permitted the identification of species with an accuracy of 95% from a dataset of 2565 fish marine resources [25,26]. The recognition of morphological features of Thunnus species using ML algorithms [27]. KNN, RF, and SVM algorithms were used to analyze the performance of the tuna outline images using the elliptic Fourier transform and deep features, and PCA of the two different morphological features was performed. Another automatic classification was implemented [28] with the phenotype textures of tuna species based on the SVM using a gray-level matrix and VGG16 to visualize texture through images. Texture feature with 83%, deep feature with 93%, and their combined feature with 95% accuracy were obtained. SVM with kernel methods is used to automatically classify the texture of tuna.

Animal species detection is carried out with the assistance of a pre-trained Darknet Coco dataset. This study was used for underwater object detection utilizing the YOLO v3 model and Fast RCNN [12] without extensive image preprocessing. Faster RCNN achieves 80.5% accuracy on the QUT fish dataset, surpassing the SSD model with 49.2% accuracy [29]. To have a better understanding of fish species interacting with temporal information of body length, width, and height [30], autonomous long-term monitoring was conducted around fish farms using the Lifeclef 2015 benchmark dataset with 91.5% accuracy [31]. Yolov8 employed a CNN by utilizing color adjustment techniques to increase the diversity of fish detection and reduce the risk factor of losing information, thereby improving with 93% accuracy [32]. The technique for understanding the characteristics of fish patterns is done by utilizing transfer learning coupled with ResNet 50 with data augmentation [33]. A method of sparse representation-based classification was proposed for the recognition and verification of fishes which maximizes the probability of partial rankings and thus obtained a 98.7% accuracy rate with a hybrid approach [34]. CNN performs a convolution process to find the association among the same group feature and is trained via back-propagation of gradients using synthetic data [35], avoiding the need for a large amount of training dataset [36], followed by binary hashing in spatial pyramid pooling used as a feature to extract information of large size image [37]. CNN classification aims to observe the fish overlapping, patterns, and correspondence in shape among fish of different species on LifeCLEF 2015 and FRGT datasets, achieving an accuracy of 98.9% [38].

To have a better understanding of how wild farm fish interact, autonomous long-term monitoring was conducted around fish farms, introducing the Nor Fisk dataset, which is publicly available at an underwater dataset accuracy of 79%, comprising YOLOv3 [39]. Inception, Concatenated ReLU, and Hypernet are some of the building blocks used by Convolution layers. The structure can be changed on the foreign fish Image collection dataset, which achieved a state-of-the-art fish detection accuracy of 99.5% [40]. To improve classification results, the fusion method where the initial layer comprises a group of classifiers tailored to specific descriptors, while the subsequent layer's classifier takes input from the first layer's scores. By employing CNN incremental learning on the Life Clef 2015 dataset, an accuracy of 81.83% is achieved [41]. Extending to videos, BRUVS was introduced to isolate objects moving, cropping, and categorizing them individually using CNN [42]. Another study [43] proposed (AlexNet) based model that achieved 98.35% for classification tested on fish species in a laboratory environment, rather than a natural environment [44]. Deep R-CNN networks provide an automatic approach for detecting and localizing fish occurrences in unconstrained underwater films with varying degrees of scene complexity. VGGNet is used to combine input features that are motion-based on raw greyscale video frames based on the information of shape and texture. A hybrid method was adopted based on optical flow and GMM detecting accuracy of 79.02% [45].

The fish counting approach based on local regression and image density of an image is achieved using image processing tools that are used to differentiate the fish area from the top-view to extract features. To make the fish counts more consistent and accurate, an imbalance in the dataset is removed using density grading applied to each of the sub-images. The proposed technique with YOLOv3 yielded promising results with a COD [coefficient of determination] of 0.9607, a root mean square error of 0.6105, and an MAE [mean absolute error] of 0.2985 [46]. Another study explored the possibility of integrating CNNs with appearance-based feature extraction to classify underwater fish images. Their model included enhanced classification by incorporating morphological attributes, including color patterns as examples, and color patterns and shapes. Despite that, the study pointed out its challenges to cope with the environmental variability, which implies the requirement for more robust models capable of accommodating a variety of underwater conditions [47]. Additionally, a two-step methodology based on transfer learning is coupled with a second classification model. By combining CNNs with classifiers such as Support Vector Machines and Linear Discriminant Analysis, they increased the F1-score from 0.92 to 0.95 with regard to a dataset from a fish market in Spain. However, the study had demonstrated how far model robustness would need to advance to become robust to real-world applications [48]. Furthermore, various CNN architectures, such as DenseNet121 and MobileNetV2 for underwater fish classification. MobileNetV2 gave a balance of accuracy compared to computational efficiency: 83.57% accuracy with 0.07 seconds processing of the images, whereas DenseNet121 was the one with the highest accuracy of 90.2%. It also pointed out the fact that real-time deployment models should be optimized for practical applications in resource-constrained situations [49]. An automated system based on a combination of Mask R-CNN for complex object detection and segmentation, with independent models for species classification and final length estimation. According to the results on a dataset consisting of 300,000 images of 163 species, FishNet achieved 89% top-1 classification accuracy. The issues with broader applicability limit the reliance on high-quality and annotated data [50].

Fish-Vista was presented using the dataset of around 60,000 images of about 1,900 species for tasks like species classification and trait segmentation. This tool seeks to enable biological discoveries in AI. The study makes the point that large, curated datasets could be powerful tools in improving fish species classification, but that just as large amounts of data are required to be classified reliably, so do they need to be annotated [51]. Additionally, M-MobileNet, a lightweight classifier of Indonesian marine species, was developed using a set of 37,462 images and provided a 97% accuracy. This study shows the feasibility of deep learning in real-world, resource-constrained settings and realizes that the models still need to be updated continuously to cope with new species [52]. However, MobileNetV2 was optimized by reaching a 96.83% validation accuracy while classifying marine species from images. The study presents information on dataset creation and augmentation techniques that can be used to deploy models in fisheries. Though such high accuracy may depend on dataset characteristics, further validation of other datasets is required [53].

The paper employs Multiple-Criteria Decision Analysis (MCDA) to manage risks in Pakistan's fisheries sector. It utilises quantitative techniques to rank operational, environmental, social, and economic factors risks [65]. A study identified the problems, including insufficient monitoring, unlawful fishing, and ineffective systems. In order to guarantee sustainable fisheries in the area, the case study highlights the necessity of improved resource management, regional cooperation, and stronger regulatory frameworks [66]. These studies focused on species classification of fish; it can be said that these studies have made remarkable progress with the use of deep learning. While high accuracies have been achieved, some issues need further research and support for real-time deployment, such as environmental variability and dataset diversity. The existing work has also been summarized in Table 1.

**Table 1:** Analysis of the existing studies.

Ref	Year	Technique	Model	Dataset	Results
[21]	2022	ML	SVM, RF, LR, NB, DT	Fish Dataset	Acc:99.89%
[22]	2022	ML	EfficientNet-Lite, MobileNetV2,ResNet50	Unsplash website Fish Dataset	Precision: 53%
[23]	2023	ML	KNN, MP, SVN	Self-created	Acc: 65.3%
[25]	2018	ML	MBN, MCN	Self-created	Acc: 95%
[27]	2023	ML	KNN, SVM	Self-created	Acc: 90%
[28]	2023	ML	SVM and VGG16	Self-created	Acc: 95%
[12]	2023	DL	YOLOv3	Fish4-Knowledge	Acc: 95%
[29]	2020	DL	Faster RCNN	QUT FISH	Acc: 80.4%
[31]	2020	DL	Res-Net 50, YOLO	Life Clef 2015	Acc: 95%
[32]	2023	DL	YOLOv8	Self-created	Acc: 93%
[34]	2019	hybrid	CNN, KNN, SV,	Fish4Knowledge	Acc: 93%
[54]	2016	hybrid	CNN, SVM, PCA	Self-created	Acc: 91%
[55]	2021	CV	OpenCV, YOLO	FSA	Precision:70%
[38]	2022	DL	CNN	Life Clef 2015	Acc: 80%
[39]	2021	DL	CNN	NorFisk	Acc: 79%
[41]	2022	DL	CNN	Life Clef 2015	Acc: 81%
[42]	2022	DL	YOLOv5	OZ Fish	Acc: 89%
[44]	2023	DL	CNN	Self-created	Acc: 94%
[48]	2024	Transfer Learning	CNN, SVM, LDA	Conil de la Frontera fish market dataset	F1: 95%
[51]	2024	DL	CNN	60,000 images, 1,900 species	Acc: 89%
[52]	2024	DL, TL	CNN, M-MobileNet	37,462 images [Indonesian marine species]	Ac: 97%
[49]	2025	DL, TL	CNN, DenseNet121, MobileNetV2	Underwater images	Acc: 90%

## Proposed research methodology

**Fig 1:**
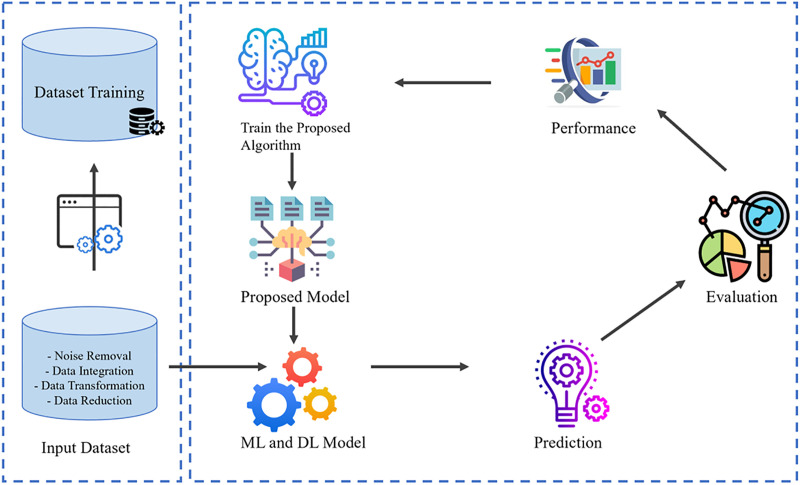
Framework flow of proposed methodology.

The proposed methodology implemented in this research work is highlighted. Data collection involves the altitude at which images were gathered. First, the fish image data that is collected is processed through preprocessing activities such as resizing, normalization, and reduction of the noise in order to achieve uniformity and enhance the quality of the model input. Then, the extraction of features is carried out with the help of the YOLOv4 architecture that applies the high-order convolutional layers and attention mechanisms and allows identifying and extracting discriminative features of several fish species effectively. These features are then extracted and subsequently used to classify, which is able to identify them correctly in different species. Lastly, the model performance is strictly measured by reference to the standard measures of Accuracy and F1-score, which are a combination of measures of overall correctness and balance between accuracy and recall, which guarantees the soundness and consistency of the proposed framework. Our research approach is based on the concept of applied research. The basic steps for the classification of Fish species include those shown in Fig 1.

- Data acquisition
- Data preprocessing
- Proposed framework
- Feature extraction
- Framework deliverables
- Detection and Classification

### Data acquisition

We used the Fish-Pak dataset, which is publicly available on Mendeley [56] and is easily accessible for research purposes. The use of specific datasets such as FiskPak is critical for enabling data-driven decisions that improve output, ecological responsibility, and economic output in the fishery sector. The dataset contains six different fish species. The species are: i) Catla, ii) Silver carp, iii) Rohu, iv) Mori, v) Cyprinus carpio, and vi) Grass carp. They shot with the Canon EOS 1300D from various pools located near the Head Qadir-Abad on the Chenab River in Punjab, Pakistan. The dataset contains a total of 915 images. The set is divided into three subclasses: i) Scale, ii) Entire Body, and iii) Head. The catla body includes 20 images, the head includes 25 images, and the scale includes 11 images. The Grass Carp body includes 11 images, 16 images, and a scale includes 9 images. Mori’s body includes 70 images, 100 images of the head, and the scale includes 71 images. Cyprinus carpio body includes 50 images, 64 images, and scale 44 images. The silver body includes 47 images, 71 images of the head, and the scale has 57 images. The Rohu body includes 73 images, the head includes 114 images, and the scale includes 62 images. For reference, we define our main dataset terms in Table 2.

**Table 2:** Description of the selected dataset.

Sr. No	Name of class	# images	Body Images	Head Images	Scale Images
1	Cyprinus carpio	158	20	25	11
2	Grass carp	36	11	16	9
3	Mori	241	70	100	71
4	Rohu	249	50	64	44
5	Silver	175	47	71	57
6	Catla	56	73	114	62
**Total**		**915**	**271**	**390**	**254**

**Fig 2:**
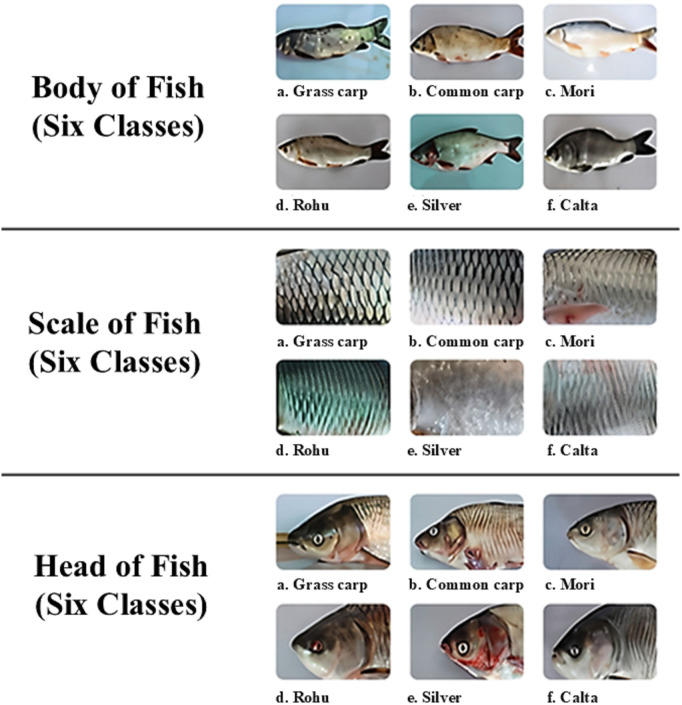
Different types of fish species image.

Fish-Pak comprises 915 images divided into six categories, as shown in Fig 2.

### Data preprocessing

Image data preprocessing is a critical step in preparing data for learning models. Data was pre-processed by eliminating redundant and irrelevant images, categorizing, and formatting them appropriately, and assuring resolution, color space, and file format uniformity. Resizing ensures uniformity, labeling maintains image categorization integrity, and data augmentation enhances dataset diversity and model robustness, but with transformations such as rotating, flipping, blurring, cropping, and others. This process has been shown to improve the performance of deep convolutional neural networks to reduce overfitting and improve model convergence. We used two types of transformations [vertical and horizontal flips]. These techniques collectively contribute to improving model performance and accuracy in image-based tasks. To recognize and filter images with labels from the Fish-Pak collection, subsets, and train the images with bounding boxes and labels for model training, we follow our proposed methodology process.

1) Annotation

Annotation involves the addition of semantic tags or labels to images within a dataset, enabling categorization, classification, and interpretation of visual content for enhancing the effectiveness of learning algorithms by providing annotated images for training and evaluation. We label certain parts of images to train the model. The YOLO v3 model was utilized, which supports files in the “txt” format or “XML” format. The spitting image and associated annotation file must be kept within the same folder.

2) Labelling

**Fig 3:**
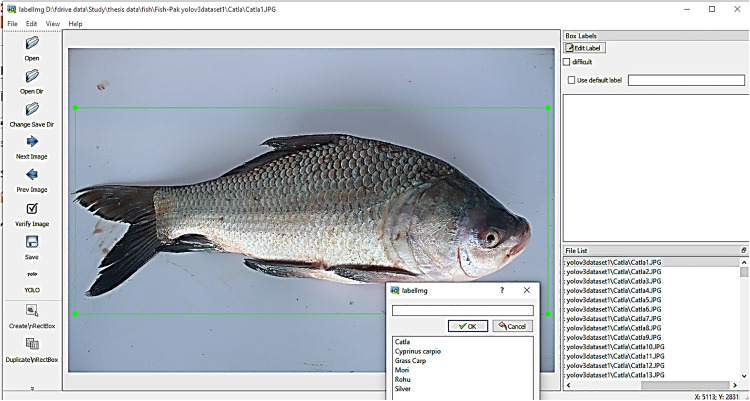
Labeling of six fish classifications, including the head types, scale types, and body shapes.

Labeling is a technique used to annotate images to enhance model training by providing clear images for learning complex patterns and relationships. Labeled datasets serve as a benchmark, ensuring the quality and reliability of datasets. Fig 3 depicts the output of our dataset labeling procedure for evaluating the performance and robustness of fish images.

3) Resizing

**Fig 4:**
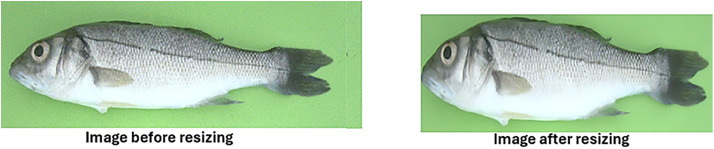
Sample image before and after resizing.

Resizing facilitates scale adjustment of image dimensions and resolution control, while preserving aspect ratios to prevent distortion. The images were resized to have dimensions 1280 × 800 for smooth training, as shown in Fig 4.

### Proposed framework

**Fig 5:**
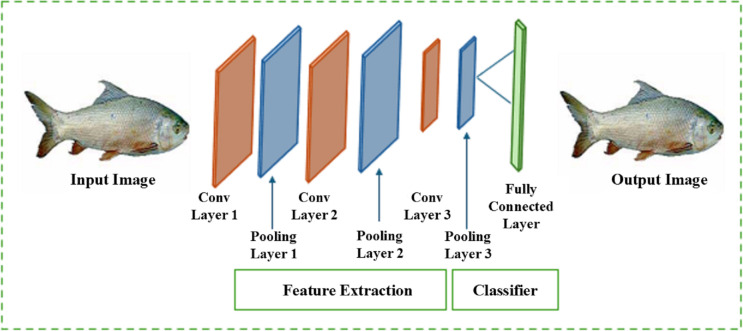
Feature extraction process by using the selected model.

In this study, we used YOLO v3 and YOLO v4 with CNN deep learning models connected to a fully connected layer coupled with features as shown in Fig 5.

**Fig 6:**
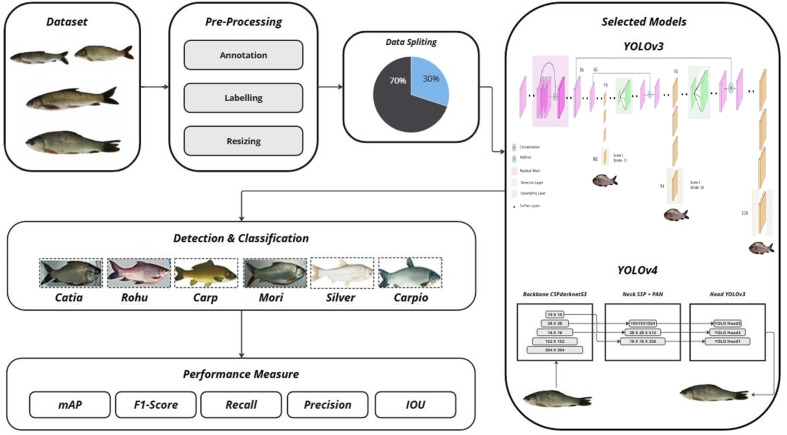
Basic steps of the proposed methodology using YOLO v3 and YOLO V4.

YOLO v3 and YOLO v4 are implemented using the darknet framework as shown in Fig 6.

Table 3 shows the machine requirements for all coding, which is done with Python 3.7.0, and the configuration is done on the local system. Python is an open-source, portable, high-level programming language. While importing libraries that are utilized in our study are: Tensor Flow, pandas, NumPy, Matplotlib, and Scikit-learn. A computer with the requirements: Intel Core i7-9700 Processor 9th Generation, NVIDIA GeForce GTX 1660Ti 6GB GDDR6, and Windows 10 (64-bit) was used for this research.

**Table 3:** System specification used for the experiments.

Parameters	Values
Machine	i7-7700k CPU
OS	Windows 10
C U D A	10.1
Cu DNN	v7.6.5
Tensor RT	6.0.1.5
TensorFlow	2.3.1

The Convolution layer applies a two-dimensional convolution on the three-dimensional input and filter to derive image locations and dimensions. The height, weight, and input channels are indicated by H1 × W1 × C. Assuming the fish image size at each margin is M and the stride is S, we can compute the height Ho and width Wo as follows in equations (1),(2).


$$\text{Ho}\;=\frac{H\text{1}-HF\;+\;\text{2}C}{S}+\text{1}$$ (1)

$$\text{Wo}\;=\frac{W\text{1}-WF\;+\;\text{2}C}{S}+\text{1}$$ (2)

After the convolution layer, the neurons in the feature map will go through a nonlinear activation function called rectified linear unit (ReLU), which is described in equation (3) as:


$$ReLU\;(x)=\;\left\{ \;\begin{array}{cc} x & \;x\geq \text{0}\\ \text{0} & \;x\leq \text{0} \end{array}\right.\;$$ (3)

To reduce computation time, pooling layers replace the outputs with ReLU.

The main purpose is to use the CNN model to yield relevant classes and labels used to extract features from images for filters. In the proposed framework, we divide the feature extraction and labeling by the combination of CNN into a 70:30 ratio for the training and testing data, inversely, then parallelly process the training classes. During the training phase, we use the softmax loss function to mutually optimize the classes and label assumptions, computed through equation (4).


$$\text{Loss}=-\frac{\text{1}}{\text{N}}\sum_{\text{i}=\text{1}}^{\text{Ni}}\left(\sum_{\text{j}=\text{1}}^{\text{Cl}}\text{1}\left({\{\text{X}}_{\text{i}}=\text{j}\;\}\text{log}\;\text{sj}\right)+\sum_{\text{k}=\text{1}}^{\text{Tl}}\text{1}\left({\{\text{Y}}_{\text{i}}=\text{K}\;\}\text{log}\;\text{sk}\right)\right)$$ (4)

Here, eq 4 is the value function. N_i_, C_1_, and T_l_ denote the total number of fish images, classes, and labels denoted by sj and sk, soft-max probabilities, respectively.

### Feature extraction and classification

For object recognition and classification, we used the YOLO classifier in our research. YOLO comes in various types, but we compared the results of the two recent algorithms: i) YOLO v3, ii) YOLO v4.

The YOLO algorithm is a standard object detection algorithm in the field of computer vision, renowned for its efficiency and accuracy, with its various versions updated till now. In addition, specific ideas have been dropped due to the algorithm's failure to achieve the requisite performance and accuracy. YOLO processes the whole image in one pass into a grid cell, predicting bounding boxes and class probabilities immediately. This method allows for extremely fast real-time object detection on videos and images, making it ideal for applications such as autonomous driving, surveillance, and object tracking. Its ability to balance speed and accuracy has been made. As a result, YOLO has become one of the most sophisticated object identification algorithms and is utilized for practical implementations. To achieve state-of-the-art results, we are comparing the two versions with their implementation and configuration.

The initial architecture, featuring 24 convolutional layers and two fully connected layers, utilized batch normalization and leaky ReLU activation but is now considered outdated for implementation due to its limited feature set. A subsequent version was developed to enhance accuracy and processing efficiency by incorporating batch normalization, anchor boxes, and an improved classifier. The third iteration leverages Darknet-53, a neural network with 53 features for extraction.

The data in the two previous versions of YOLO, after training with the Darknet feature extractor, was passed through additional layers before being used for predictions in the final stages of the object detector. In YOLO v3, a significant change was made by adding prediction layers to the side network instead of stacking them at the end. YOLO v3's key feature is its ability to detect objects at three different scales, achieved by creating three distinct scale detectors using features from the last three residual blocks.

The output of YOLO v3 is a tensor with a shape of [S × S × Number of filters], where the number of filters varies based on the specific implementation, as in equation (5).


Filters =[classes+Cp+Xc+Yc+W+H x B] (5)

Where Cp is the confidence of existing species in the grid cell result, X and Y are the box coordinates, and W, H, and B are the width, height, and no. of boxes represented, respectively.

The number of filters in the last YOLO layer is adjusted based on the number of classes being detected, as described in equation (6). In this case, with one class of fish being detected, the number of filters is set to 18. This relationship between the number of classes and the number of filters allows for efficient detection and classification within the YOLO architecture.


Filters = (classes+5)*3 (6)

Our requirements should be reflected in the configuration file shown in Table 4. The batch size sets the training batches. Throughout the training, the neural network is modified iteratively depending on mistakes made on a training dataset. It's impossible to update the weight with all the images at the same time. As a result, the batch size is a collection of images used in an iteration. Darknet subdivision parameters are set to a multiple of 2, utilizing 32 filters at 100 batches until the training is finished with a lr of 0.01 to 0.0001, momentum 0.9 over 100 epochs.

**Table 4:** Implementation parameters for both architectures.

Parameters	YOLO v3 Values	YOLO v4 Values
Batch	4	4
Subdivisions	4	4
Width	416	416
Height	416	416
Channel	3	3
Learning rate	0.01	0.01
Steps	9600, 10800	9600, 10800
Classes	6	6
Filters	32	32

The pre-trained weight models Darknet53.74 and YOLOv4 conv.137 were utilized in YOLO v3 and YOLO v4, incorporating features like CSP Darknet-53, SSP block, PANet, and a detection head. Both YOLO v3 and YOLO v4. To prevent overfitting, a unique validation set separate from the training and testing datasets was employed. This work introduced residual blocks, skip connections, and up-sampling techniques, significantly enhancing the accuracy of the algorithm. The backbone feature extractor CSPDarknet53 in YOLOv4 notably improved both speed and accuracy. Training was closely monitored until the average loss stabilized, and the mean Average Precision [mAP] curve reached its peak value. Graphical plots of the training iterations’ outputs were used to assess progress.

### Performance evaluation measure

Fish species detection is measured using a variety of indicators, including precision, accuracy, mAP, IoU, and F1 score, which are used to evaluate the performance of classification and object detection models. Precision is a measure of how many positive instances are correctly identified out of all the instances the model predicts to be positive; that is, it measures the skill of the model to avoid false positives. The accuracy is a measure of the percentage of correctly classified instances, which gives an overall performance measure of an object detector across all the classes. mAP is popular in object detector tasks; it is the average of the Average Precision values of each object class, and it well describes the localization as well as the classification behavior. IoU measures the overlap of the predicted and ground truth bounding boxes by the area of their union divided by the area of their intersection, which denotes the extent to which objects are localized in space. Last but not least, the F1 score, which is the harmonic mean of the precision and recall, offers a balanced measure to be used especially when there is a class imbalance. Taken together, these measures provide an overview of the accuracy of a model, its localization power, and its strength. The efficacy of datasets and prediction models is evaluated using these parameters, evaluating a model’s performance on a dataset, and key metrics are shown by following equations (7–11). In the assessment of the model’s performance on a dataset, to measure key metrics are Accuracy, Precision, Recall, F1 Score, and Mean Average Precision (mAP):


$$\text{Accuracy}\;=\frac{TP+TN}{TF+FN+FP+TP}$$ (7)

$$\text{Precision}\;=\frac{TP}{TP+FP}$$ (8)

$$\text{Recall}\;=\frac{TP}{TP+FN}$$ (9)

$$\text{F1}-\text{score}\;=\frac{\text{2}[Precision*Recall]}{Precision+Recall}$$ (10)

mAP = 1 N ∑ APi (11)

## Results and discussion

**Fig 7:**
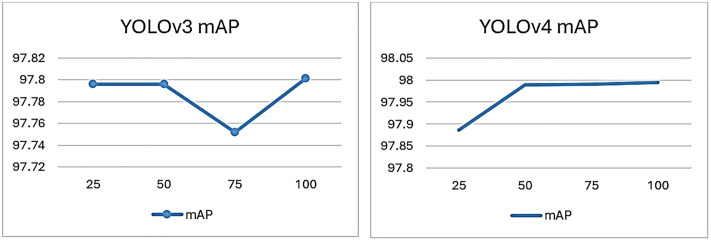
YOLOv3 and YOLOv4 training mAP Chart.

The results are divided into targeted species classes and model performance with the effect of an increasing altitude, and lastly, sample test image results. The performance is determined by analyzing our proposed YOLO v3 and YOLO v4 models, as depicted in Fig. 7.

For YOLOv3, the mAP values exhibit slight fluctuations around the 97% mark. Notably, there is a visible dip in performance at the 75-epoch mark, where the mAP drops below 97%, suggesting a temporary decline in detection accuracy, potentially due to overfitting training data at this point. On the other hand, YOLOv4 demonstrates a more consistent and improved performance. Starting from around 97% at 25 epochs, the mAP steadily increases and stabilizes around 98% as the epochs progress to 100. This indicates that YOLOv4 generally provides better and more stable detection accuracy for fish species compared to YOLOv3, possibly due to enhancements in the architecture and training methodologies of YOLOv4. Overall, the YOLOv4 model appears to be suitable for fish species detection based on the performance evaluation mAP metrics given.

**Fig 8:**
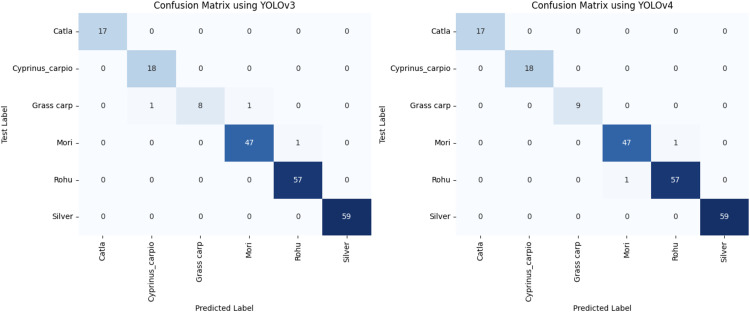
YOLOv3 and YOLOv4 confusion matrix.

Fig 8 presents the confusion matrices for YOLOv3 and YOLOv4 models, comparing their performance in detecting fish species after 100 epochs. The YOLOv3 model shows high accuracy, correctly identifying most species with minor misclassifications, such as one instance of Cyprinus carpio misclassified as Grass carp. The model YOLOv4 demonstrates similar performance, with slightly improved consistency across species, maintaining correct classification for all instances without misclassification. This comparison indicates that while both models perform well, YOLOv4 offers marginally better precision in fish species detection.

**Fig 9:**
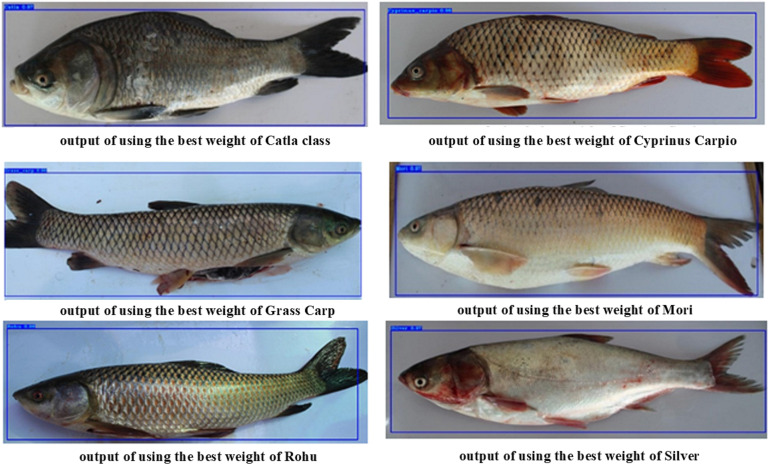
Resultant images after using the proposed models.

The following Table 5 lists the values of mAP, F1-Score, Recall, Precision, and IOU at the epochs of 25,50,75, and 100. These values are obtained by applying YOLO v3 to the test in the dataset. On images, we train and test proposal region and object detection networks to classify the Fish species by combining their Head, body, and scale images. The total training iterations are 100. We will show the values of all parameters after every 100 iterations. The following Table 6 lists the values of mAP, F1-Score, Recall, Precision, and IOU at the epochs of 25,50,75, and 100. YOLOv3 demonstrates high performance across all iterations, maintaining an mAP of around 97% to 98% with a perfect f1-score at most iterations, except for a dip at 50 iterations. In contrast, YOLOv4 consistently achieves higher mAP values, peaking at 99% with slightly lower but stable F1-scores and precision across all iterations. Both models exhibit similar Recall and IOU values, but YOLOv4’s marginally higher mAP suggests it is more effective in detecting fish species with top accuracy. The classified images showing the specific class species are shown below by highlighting them in a blue square with the class name at the top left-most corner. This shows that the inland fish species can be classified and detected to a specific class by utilizing their Head, scale, and body region details shown in Fig. 9. The figure shows performance curves of YOLOv3 and YOLOv4 fish species classification, which provide vital information on the learning behavior, generalization, and efficiency of both models. YOLOv4 has faster stable, and faster convergence than YOLOv3, which implies more efficient learning and faster optimization during training. YOLOv4 features a loss curve that is much smoother, with less fluctuation and better weight updates, as well as less susceptibility to overfitting. This means that the advanced architectural modification in YOLOv4's CSPDarkNet53 backbone and Mish activation facilitates better acquired and propagated features and better generalization. However, YOLOv3's performance is quite good, although the loss goes down at a relatively slow pace, showing that it takes longer for it to achieve the best performance. Also, YOLOv4 has a steeper rise in the accuracy curve compared to YOLOv3, achieving a higher accuracy at a plateau. This implies better classification precision and recall. The minimal gap between the training and validation curves in YOLOv4 is deemed to exhibit reduced overfitting, which is further proof of its robustness for dealing with hierarchical fish species variations. Additionally, YOLOv4 outperforms YOLOv3 in terms of mAP [99.657% to 63.317%], which shows that YOLOv4 can classify fish species with more confidence compared to YOLOv3 with fewer false positives. Analysis of these results confirms that the YOLOv4 model has a better overall detection accuracy and computational efficiency than YOLOv3; therefore, the YOLOv4 model is a better model for use in fisheries applications where specific and automated species recognition is critical.

**Table 5:** Comparison of the results of YOLO V3 using different epochs.

Epochs	mAP	F1-Score	Recall	Precision	IOU
25	97.886	1.00	1.00	1.00	0.75
50	97.989	0.98	1.00	0.99	0.86
75	97.991	1.00	0.98	0.99	0.87
100	97.995	1.00	1.00	1.00	0.86

**Table 6:** Comparison of the results of YOLO v4 using different epochs.

Epochs	mAP	F1 – Score	Recall	Precision	IOU
25	98.688	0.97	0.97	0.99	0.75
50	99.656	0.98	0.99	0.98	0.87
75	98.144	0.99	0.99	0.99	0.89
100	99.657	0.99	0.99	0.99	0.89

Ablation study of the YOLO model offers a profound insight into how different architectural components contribute to the gain in fish species classification performance. The study also points out that YOLOv4 extensively refines the features extracted by the network using refinement and spatial attention mechanisms, as well as a CSPDarkNet53 backbone, compared to YOLOv3. Results of the experiments show that YOLOv4 outperforms in terms of Precision, Recall, and mAP compared to other researched models due to a high level of fish species variations. Therefore, the study also evaluates the effects of many hyperparameters, input resolutions, and training strategies, and confirms that higher resolution inputs and optimized anchor box configurations lead to better detection performance. This confirms that YOLOv4’s architectural refinements have a non-negligible contribution in terms of false positives and false negatives reduction, pushing the model towards a more robust one faced with real-world applications of fish species classification.

With a remarkable accuracy of almost 97%, the proposed Fish YOLO v4 model performed better than practically all the other studies earlier, and the classification accuracy is also increased and guaranteed more than before, as displayed in Table 7. The proposed model performed better than other models, such as ResNet-50 with YOLO [2020] and YOLOv5 [2022], which reached accuracies of 95% and 89%, respectively. Furthermore, the YOLOv4-based model surpassed the YOLO OpenCV version [2021] with a precision of 70%. The proposed method is fairly close to competitive results, though in terms of accuracy [97% from CNN with M-Mobile Net 2024 model], to more recent models of CNN with M-MobileNet [2024]. Also, it outperforms DenseNet121 and MobileNetV2 [2025], which reported 90% accuracy, by a margin, indicating relative superiority for running in real time and deployment efficiency. Consequently, the way to go forward is to determine whether the model works across many and large enough datasets, as well as diverse ones.

**Table 7:** Comparisons of the results of YOLO v4 with the existing research work.

Ref	Year	Technique	Model	Dataset	Results
[24]	2019	DL, TL	CNN	Fish Pak	Acc: 95%
[38]	2020	DL	Res-Net 50, YOLO	Life Clef 2015	Acc: 95%
[55]	2021	CV	OpenCV, YOLO	FSA	Precision:70%
[50]	2022	DL	YOLOv5	OZ Fish	Acc: 89%
[52]	2023	DL	CNN	Self-created	Acc: 94%
[52]	2024	DL, TL	CNN, M-MobileNet	37,462 images (Indonesian marine species)	Ac: 97%
[49]	2025	DL, TL	CNN, DenseNet121, MobileNetV2	Underwater images	Acc: 90%
Proposed		DL	YOLO v4	Fish Pak	Acc: 97%

## Conclusion and future work

The study focuses on automated fish detection in submarine environments using deep learning algorithms. YOLO-V4 outperforms YOLO-V3 in accuracy and training speed. The pattern of YOLO-V4 and CNN models yields precise and accurate detection to achieve real-time performance with high precision and robustness by using the Fish Pak dataset. In this study, a new approach was introduced involving a two-layer classifier designed to categorize fish based on their species. Various preprocessing techniques were applied to the datasets to mitigate overfitting and enhance detection accuracy. Experimental findings demonstrated that YOLOv4 achieved the highest accuracy of 97.801% on the training dataset and 97.25% on the testing dataset. The proposed methodologies will be used in real-time for multiple on-land fish species detection. The use of a relatively small dataset with only six fish species is one limitation of the system that might limit the ability of developing the model to generalize to a wider range of different species. The model can be made robust and applicable in practice if the dataset is expanded with more diverse categories of fish and larger sample sizes. In the future, we aim to enhance our work by creating a large dataset that contains more classes of Fish and different kinds of their species and subcategories in their growth life cycle, i.e., larva, pupa, and adult, by applying the latest version of YOLO, such as v5 and v7.

The proposed framework for fish species classification has a number of practical implications across multiple sectors. It can help in automating separation and packaging processes, which are currently manual and labor-intensive. By enabling the automatic identification and sorting of fish species at processing facilities, this framework has the potential to reduce reliance on human labor while improving both accuracy and operational efficiency [57]. In addition, it can help ensure accurate product labeling and reduce the risk of species misidentification or fraud. Marine biologists can benefit from automated species recognition, which facilitates large-scale biodiversity assessments and ecological research. The proposed framework can also guide species-based feeding, health monitoring, and stock management in aquaculture. While the proposed framework demonstrates high accuracy under controlled imaging conditions, its applicability to real-world rapid assessment scenarios may be affected by lower image quality and variability. To address this, future work will focus on enhancing model robustness through transfer learning techniques and extensive data augmentation, enabling effective deployment in diverse field environments. Furthermore, the model can be integrated into mobile applications for educational use, assisting fishermen, students, and citizen scientists with on-the-go species identification. Given the regional specificity of the training data, the framework can also contribute to national fisheries policy and operations. It enables automated species tracking, strengthens regulatory enforcement, and provides data-driven insights for improving transparency and efficiency in the fishing industry.
